# Identification of Gene Modules and Hub Genes Involved in Mastitis Development Using a Systems Biology Approach

**DOI:** 10.3389/fgene.2020.00722

**Published:** 2020-07-13

**Authors:** Mohammad Reza Bakhtiarizadeh, Shabnam Mirzaei, Milad Norouzi, Negin Sheybani, Mohammad Sadegh Vafaei Sadi

**Affiliations:** Department of Animal and Poultry Science, College of Aburaihan, University of Tehran, Tehran, Iran

**Keywords:** weighted gene co-expression network, protein-protein interaction, RNA-seq, hub genes, bovine

## Abstract

**Objective:**

Mastitis is defined as the inflammation of the mammary gland, which impact directly on the production performance and welfare of dairy cattle. Since, mastitis is a multifactorial complex disease and the molecular pathways underlying this disorder have not been clearly understood yet, a system biology approach was used in this study to a better understanding of the molecular mechanisms behind mastitis.

**Methods:**

Publicly available RNA-Seq data containing samples from milk of five infected and five healthy Holstein cows at five time points were retrieved. Gene Co-expression network analysis (WGCNA) approach and functional enrichment analysis were then applied with the aim to find the non-preserved module of genes that their connectivity were altered under infected condition. Hub genes were identified in the non-preserved modules and were subjected to protein-protein interactions (PPI) network construction.

**Results:**

Among the 25 modules identified, eight modules were non-preserved and were also biologically associated with inflammation, immune response and mastitis development. Interestingly most of the hub genes in the eight modules were also densely connected in the PPI network. Of the hub genes, 250 genes were hubs in both co-expression and PPI networks and most of them were reported to play important roles in immune response or inflammatory pathways. The blue module was highly enriched in inflammatory responses and *STAT1* was suggested to play an important role in mastitis development by regulating the immune related genes in this module. Moreover, a set of highly connected genes were identified such as *BIRC3, PSMA6, FYN, F11R, NFKBIZ, NFKBIA, GRO1, PHB, CD3E, IL16, GSN, SOCS2, HCK, VAV1* and *TLR6*, which have been established to be critical for mastitis pathogenesis.

**Conclusion:**

This study improved the understanding of the mechanisms underlying bovine mastitis and suggested eight non-preserved modules along with several most important genes with promising potential in etiology of mastitis.

## Introduction

Mastitis, inflammation of the mammary gland, is one of the most prevalent diseases in dairy cattle around the world (Munro et al., 1984; Biggs, 2009). Mastitis is mainly caused by pathogens, which can be divided into contagious, non-contagious (*Staphylococcus aureus, Streptococcus agalactiae, Corynebacterium bovis, Mycoplasma bovis*) and peripheral (*E. coli, Streptococcus dysgalactiae, Streptococcus uberis*) (Zhao and Lacasse, 2008). Mastitis has been considered as one of the most economically important disorders due to its negative effects on quantity (Schukken et al., 2009) and quality of milk (Hortet and Seegers, 1998), animal welfare (Peters et al., 2015) reproductive performance (Kumar et al., 2017), increased use of antibiotics (Groot and van’t Hooft, 2016) and the need for the treatment and premature culling of dairy cows (Heikkilä et al., 2012). *Streptococcus uberis* enters the udder via the teat canal and produces clinical and subclinical cases of mastitis (e.g., Hogan et al., 1989; Williamson et al., 1995). In this regard, environmental streptococci *(Streptococcus uberis)* are responsible for one third of clinical mastitis cases (Hillerton and Berry, 2003) and considers as the most prevalent mastitis causing pathogens throughout Europe and North America (Ward et al., 2009; Reinoso et al., 2011). In comparison to *Escherichia, Streptococcus uberis* induces a delayed mRNA expression of *interleukin-8* by epithelial cells (Wellnitz et al., 2006). This cytokine is involved in the recruitment of neutrophils, which play roles in the healing of intramammary infections (Barber and Yang, 1998). Previous studies reported that a wide variety of strains can infect the mammary gland with different intensity (Leigh et al., 1990; Phuektes et al., 2001). This constitutes a major obstacle in the effective treatment and development of strategies to control this important mastitis pathogen. Hence, a more precise identification of dynamics of infection and new candidate genes in the development of mastitis induced by Streptococcus uberis would be useful.

Several studies have been conducted on different aspects of the topic such as nutrition (Heinrichs et al., 2009), management (Neave et al., 1969), or genetic (De Vliegher et al., 2012) to prevent or alleviate the consequences of bovine mastitis. The previous studies have been reported some differentially expressed genes (DEGs) as potential candidates in both inflammatory responses (Lutzow et al., 2008) and overall metabolism (Mitterhuemer et al., 2010) including *TLR2, TLR4, S100A12, IL8, CD14, IL-1*β, *IL-6, IL-8*, and *TNF*α. For example, Lawless et al. (2014) used RNA sequencing to identify mRNA and miRNA genes involved in bovine mastitis and reported more than 3700 DEGs, which were significantly enriched in inflammatory and non-glycolytic metabolic pathways. However, it is well-known that differential expression analysis merely focuses on the effect of individual genes rather than considering the effect of clusters of genes. Therefore, individual assessment of gene expression may fail to explain the complex etiology of diseases or traits of interest.

On the other hand, gene expression data can also be used for constructing the gene regulatory networks (like co-expression gene networks), using a system biology approach, to better understand molecular mechanisms behind the complex diseases such as mastitis (Nielen et al., 1995). Moyes et al. (2009) used a gene regulatory approach to understand the most affected gene networks in bovine mammary tissue in response to infection. They found some pro-inflammatory pathways associated with a marked inhibition of lipid synthesis, stress-activated kinase signaling cascades and *PPAR* signaling were activated (Moyes et al., 2009). In the study of Han (2019) by using gene regulatory network approach, discovered that differential expressed genes in the *E. coli*-inoculated and the *S. aureus*-inoculated groups, were associated with the RIG-I-like receptor signaling pathway and lysosome pathway, respectively. The main assumption underlying gene co-expression networks states that highly co-expressed genes are likely to be functionally associated. In this regard, a well-known and widely used method for constructing the gene co-expression networks is weighted gene co-expression network analysis (WGCNA) (Langfelder and Horvath, 2008). WGCNA considers the differences in the response of the samples at different time points by clustering the genes into the specified modules based on the expression correlation patterns among genes across the samples. Potential of this approach for grouping genes into the functional modules and revealing regulatory mechanisms underlying the complex traits have been highlighted in many recent studies (Bakhtiarizadeh et al., 2018). Using WGCNA, highly connected genes (called hub genes) can also be screened within the modules, based on intramodular gene connectivity. Moreover, WGCNA provides a unique network-based strategy to access whether the network density and topology pattern of a module, obtained from a given set of samples (normal samples), are preserved in another set of samples (disease samples) (Langfelder and Horvath, 2008). Thus, some modules and their hub genes that are not preserved between these situations may potentially be involved in the biological processes of interest (Botía et al., 2017).

It is of great significance to understand the mechanisms of disease. High-throughput technologies combined with novel computational systems biology approach have provided new opportunities for a better understanding of the molecular regulatory mechanisms that mastitis can be developed (Sharifi et al., 2019). Hence, in the present study, RNA-Seq data was obtained from a previous study (Lawless et al., 2014) and was used to construct the modules with biologically related genes in healthy bovine samples by WGCNA method. Then, module preservation functionality in WGCNA was served to discover non-preserved modules in infected samples and further functional analysis were carried out. The main assumption was that non-preserved modules may contain potential functionally related genes or possibly share common biological regulatory functions in pathological processes related to mastitis. This effort can accelerate discovery of genes as well as molecular mechanisms responsible for immune response to mastitis in cattle.

## Materials and Methods

### Gene Expression Dataset

RNA-Seq data of healthy and infected bovine samples were obtained from the Gene Expression Omnibus (GEO) database at the National Center for Biotechnology Information (NCBI) under accession number of GSE51858. The data included samples from milk of five infected (via the teat canal of right-front quarter with 500 units of *Streptococcus uberis* (*S. uberis0140*) colonies at 0, 12, 24, 36, 48 h after infection), as well as five healthy Holstein cows, at the same time points. More details of the data can be found in the original paper (Lawless et al., 2014). Briefly, in Lawless et al., study Milk-derived *CD14* + monocytes (*CD14* is a receptor that binds to *LPS* and mediates the LPS-induced activation of host cells) were isolated by fluorescence-activated cell sorting. These cells were then labeled with monoclonal anti-bovine *CD14* and a PE-conjugated anti-mouse *IgG1* antibody. Labeled cells were separated based on fluorescence intensity and the cells with more than 95% purity were isolated from the milk of each animal. The infection was monitored using recorded milk bacterial counts (CFU/ml) and somatic cell counts (per ml) at each of the five time points for each animal (control and infected). An Illumina HiSeq 2000 tool was used to generate 50-bp single-end reads and totally 50 samples were created (five biological replications for each time point). After obtaining the data, five samples (including GSM1254091, GSM1254117, GSM1254119, GSM1254120, and GSM1254121) were removed due to low quality reads (*Q* < 20 and low number of reads) and the remaining 45 samples (24 healthy and 21 infected samples) were kept for further analysis.

### RNA-Seq Data Analysis and Preprocessing

Quality control of the raw data was evaluated using FastQC (version 0.10.1) (Andrews, 2010). Trimmomatic software (version 0.32) (Bolger et al., 2014) was used to filter out the adapter sequences and low quality bases/reads with trimming criteria: LEADING:20, ILLUMINACLIP: Adapters.fa:2:30:10, and MINLEN:25. The clean reads were checked again using FastQC. The clean reads were then aligned to the reference bovine genome using Tophat software (version 2.1.0) (Trapnell et al., 2009). The bovine genome was downloaded from the Ensembl database (version UMD_3.1). The reads were mapped according to the genomic annotations provided in the bovine Ensembl annotation in gene transfer format (GTF). HTSeq-count software (Python package HTSeq, version 2.7.3) (Anders et al., 2015) was applied to count aligned reads that overlapped with all bovine gene using the bovine GTF file. All the count files were then merged into a count table containing read-count information for all samples. Since WGCNA approach was originally developed for microarray data, raw counts data have to be normalized to be suitable for WGCNA analysis. Hence, the raw counts data were normalized to log-counts per million (log-cpm), using the voom normalization function of the limma package (version 3.40.2) (Smyth, 2005). Taking into account that genes with very low expression are less reliable and indistinguishable from sampling noise, the genes with less than one cpm (count per million) in at least five samples and standard deviation lower than 0.25 were filtered out.

### WGCNA Network Analysis

Network analysis was performed according to the protocol of the WGCNA R-package (version 1.68) (Langfelder and Horvath, 2008). Firstly, in order to remove outlier samples, distance-based adjacency matrices of samples were estimated and sample network connectivity according to the distances was standardized. Samples with connectivity less than -2.5 were considered as outliers and were excluded (Supplementary File S1). Then, reliability of samples and genes for WGCNA analysis was inspected to exclude the samples and genes with excessive missing entries and genes with zero variance. Based on the assumption that non-preserved modules between healthy and infected groups may be functionally related to mastitis, healthy samples were considered as the reference set for module detection. Here, a signed weighted gene co-expression network was constructed, which only consider positively correlated genes and genes with negative correlation are considered unconnected. Generally, signed networks are preferred over unsigned networks and appeared to be more robust by identifying modules with more significant enrichment of functional terms (Langfelder and Horvath, 2008; Smita et al., 2013). Also, biweight midcorrelation was used instead of the Pearson or Spearman correlation, because it is robust and resistant to outliers (Song et al., 2012). To be sure that the constructed network satisfies the scale-free topology (a fundamental property of biological gene networks in which some genes are more connected than others), an appropriate soft-thresholding power was calculated by plotting the R^2^ (scale-free topology fitting index) against soft thresholds. At β = 23, network created by WGNCA showed >90% scale free topology in healthy samples. Supplementary File S2 shows the relationship between the β and scale free topology fitting index in healthy samples. Then, the co-expression modules were constructed using automatic module detection function blockwiseModules of WGCNA and four important steps were performed including (1) create weighted adjacency matrix by calculating biweight midcorrelation between each gene pairs to determine connection strengths between them, (2) transform weighted adjacency matrix into a topological overlap matrix (TOM), which summarizes the network connectivity of genes, (3) identify the modules by average linkage hierarchical clustering analysis through a dynamic hybrid tree cutting algorithm and using TOM graphs as input (by defining a dissimilarity matrix, 1-TOM), (4) merge the modules with highly correlated eigengenes, which have extremely similar expression profiles. The following parameters were used; power = 23, corType = “bicor,” minModuleSize = 30, mergeCutHeight = 0.25, maxBlockSize = 17,000, reassignThreshold = 0, networkType = “signed,” and TOMType = “signed.”

### Preservation Analysis

Module preservation analysis allowed us to evaluate how well the modular structure of the healthy samples are preserved in the infected samples. To do this, the function “module Preservation” of WGCNA package was used and a permutation test (based on the generation of 200 random permutations) was calculated, which assesses the preservation of the connectivity and density of each network module. In this study, a combination of two widely used network-based module-preservation statistics including Zsummary and medianRank scores were applied to determine the modules which are preserved, semi-preserved or non-preserved. Zsummary is a composite module preservation statistic that simultaneously assess whether the genes in a defined module in the healthy samples remain highly connected in the infected samples as well as investigate whether the connectivity patterns between the genes in the healthy samples remain similar, compared with the infected samples (Langfelder et al., 2011). Zsummary allows for significance thresholds but shows a strong dependence on module size and tends to increase with module size. On the other hand, medianRank is the mean of median ranks computed for connectivity and density measures of each module and is independent of module size. Hence, medianRank is more robust and always apply to confirm the Zsummary results. Totally, a module with lower medianRank or higher Zsummary tends to exhibit stronger preservations. Here, the modules with Zsummary >10 and medianRank <8 were interpreted as highly preserved, the modules with 2< Zsummary ≤8 and medianRank <8 were defined as semi-conserved and the modules with Zsummary <2 and medianRank ≥8 were considered to be non-preserved.

### Functional Enrichment Analysis

In order to better understand the potential mechanisms of how module genes can affect mastitis, all genes in the modules as well as their hub genes were separately subjected into gene ontology (GO) and KEGG pathways analysis using Enrichr online tool (Chen et al., 2013). Only terms with adjusted *p* < 0.05 (FDR by Benjamini–Hochberg method) were considered.

### Potential Hub Genes Identification and PPI Network Construction

The genes with the highest degree of connectivity in the module are considered as the hub genes and is expected to exhibit higher biologically significance compared with the other gene members of the module. Module membership or kME is defined as the correlation between the gene expression profile and the module eigengene for each gene in the module. In other words, kME measures the connection strength of a gene with the module it has been assigned to and to the other modules as well. We used kME >0.7 to identify the hub genes in the non-preserved modules. Next, the identified hub genes in each module were subjected to PPI network construction using Search Tool for the Retrieval of Interacting Genes (STRING) database and medium stringency settings was set and included all possible interactions (Szklarczyk et al., 2019). This analysis investigates whether co-expressed hub genes in each module are still significantly associated, based on PPIs data, or not. To explore the important nodes and subnetworks in the PPI networks, cytoHubba application (version 0.1), a Cytoscape plugin, was used (Chin et al., 2014). This application suggests 12 topological analysis methods for ranking the important nodes in a biological network including maximum click centrality (MCC), density of maximum neighborhood component (DMNC), maximum neighborhood component (MNC), degree method, edge percolated component (EPC), bottleneck, EcCentricity, closeness, radiality, betweenness, stress, and clustering coefficient (Chin et al., 2014). The top 50 important genes in each PPI network were ranked using all methods. To establish a consensus rank of the important genes, rankAggreg R package (version 0.6.5) (Pihur et al., 2009) was applied based on two methods including cross-entropy Monte Carlo algorithm and Genetic algorithm. Finally, the overlapped genes between these two methods were defined as highly connected genes. Cytoscape (version 3.7.2) (Saito et al., 2012) was used to visualize the gene co-expression network of the important non-preserved modules.

## Results

### RNA-Seq Data Analysis

**FIGURE 1:**
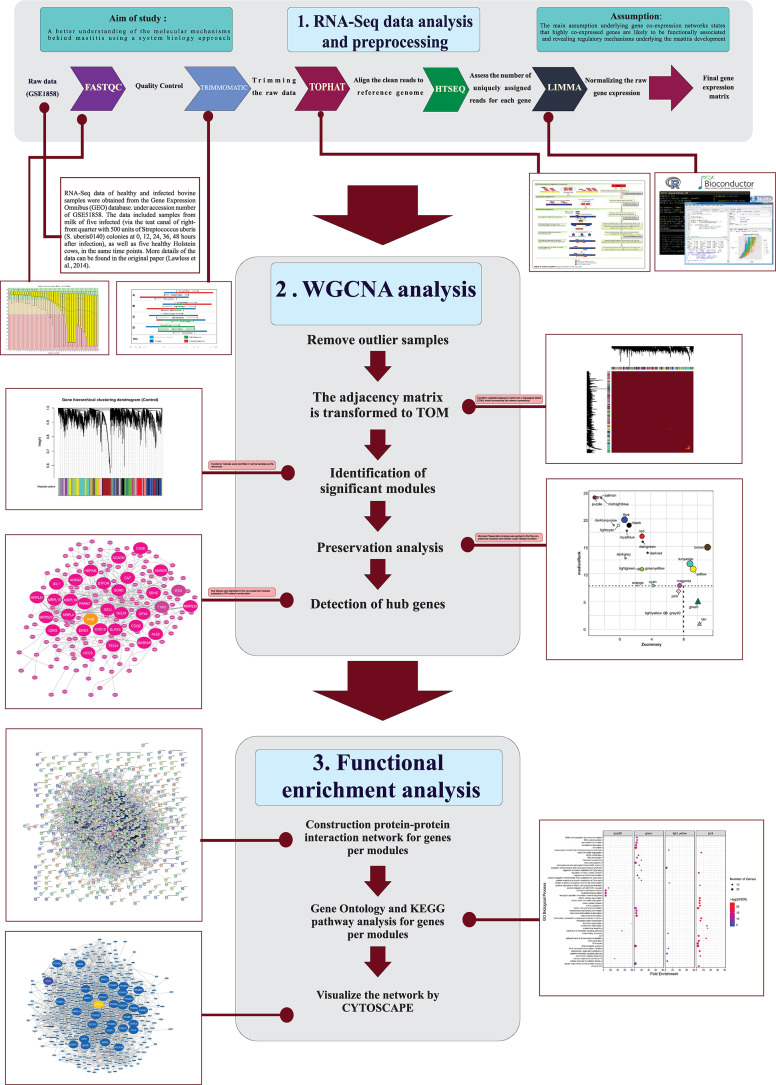
The used pipeline for construction of the co-expression gene network.

A stringent stepwise pipeline was used to construct the co-expression gene network (Figure 1). A total of 1,935,472,920 reads related to 45 RNA-Seq samples (24 healthy and 21 infected bovine samples) were analyzed. After trimming the raw data, a total of 1,690,493,091clean reads were obtained. The average sample sizes were 43 and 37 million reads before and after quality control, respectively. On average 93% of the clean reads were aligned to reference genome (ranged from 84 to 94%). The summary of the RNA-Seq data and mapping of all samples are provided in Supplementary File S3. To remove the genes with very low expression in most of the samples, different filtering parameters were used and a total of 9,721 genes were remained for network construction. The complete list of these genes along with their log-transformed expressions are provided in Supplementary File S4.

### Weighted Co-expression Network Construction

**FIGURE 2:**
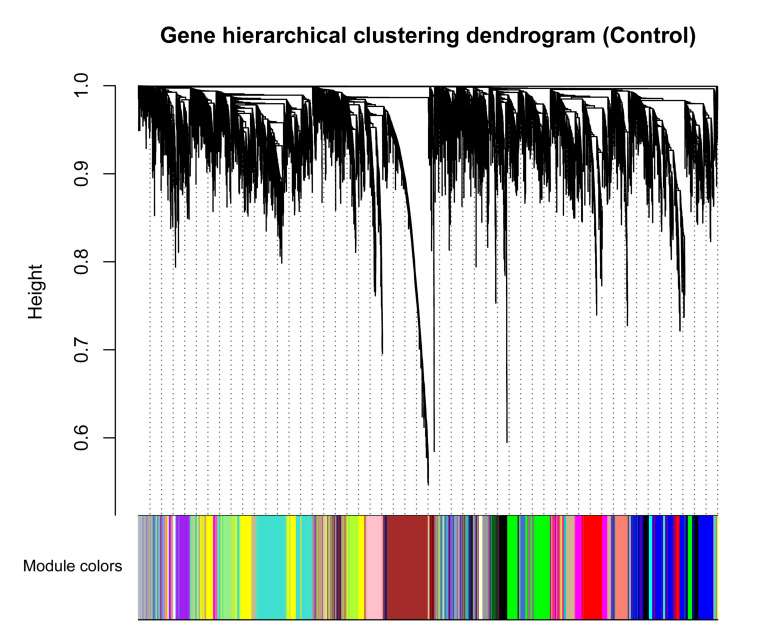
Clustering dendrogram of genes based on a dissimilarity measure (1-TOM), which was then used to group genes into 25 modules in healthy samples. The branches correspond to modules of highly interconnected groups of genes. The height (*y*-axis) indicates the co-expression distance and the *x*-axis corresponds to genes. Colors represent the 25 different modules along with gray indicating genes that could not be assigned to any module.

To avoid the influence of potential outlier samples, one outlier sample (GSM1254086, from healthy samples) whose connectivity was less than -2.5 was removed. To fulfill the criteria of approximate scale-free topology, the soft threshold power beta was set to 23 in which R^2^ was >0.9 (Supplementary File S2). Taking healthy bovine samples as the reference set, hierarchic clustering and dynamic branch cutting procedures resulted in identification of 25 modules. Each module as a branch of the resulting clustering tree was labeled by a unique color (Figure 2). The modules inferred showing different sizes, with an average of 398 genes. The turquoise module had the largest number of genes (1,132), while the orange module with 42 genes showed the smallest number of genes (Supplementary File S5). Also, 460 genes were assigned into gray module, which represents the genes that were not co-expressed based on gene dissimilarity measure and were not assigned into any of the modules.

### Module Preservation Analysis

**FIGURE 3:**
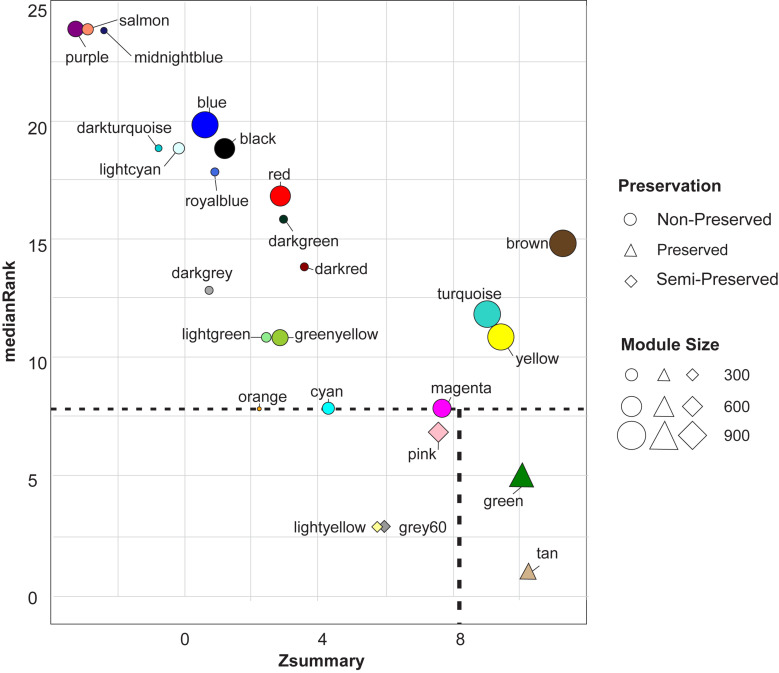
The medianRank (left scatter plot) and Zsummary (right scatter plot) statistics of the module preservation. The medianRank and Zsummary of the modules close to zero indicate the high and low degree of module preservation, respectively. A negative Zsummary value indicates the modules’ disruption.

Module preservation analysis revealed two highly preserved modules including green and tan modules. Three modules were found to be semi-preserved including pink, grey60, and light-yellow modules. Preservation analysis indicated that expression patterns and network characteristics among the co-expressed genes of 20 modules were changed during the physiological state alteration (healthy state to mastitis state) (Figure 3). Of these, purple and salmon modules were detected as the least preserved modules with 391 and 280 genes in each module, respectively. Their Zsummary were -3.1 and -2.9, respectively, and medianRank score was 24 for both (Supplementary File S6 and Figure 3).

### Functional Enrichment Analysis

**FIGURE 4:**
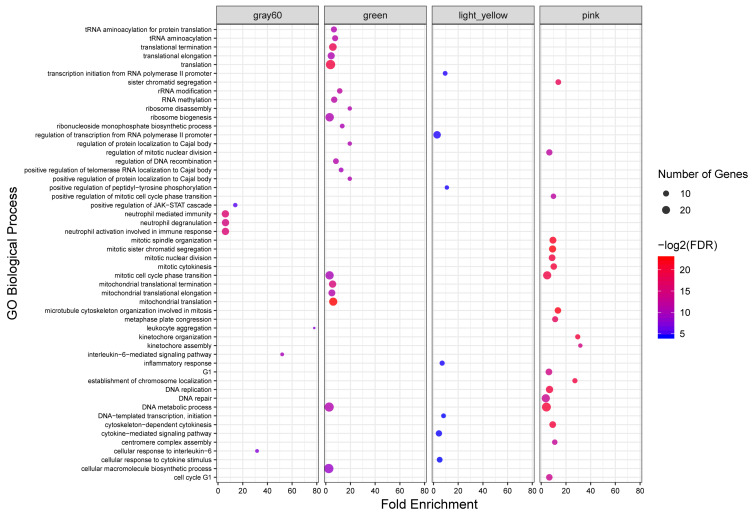
GO analysis results of highly and semi-preserved modules. Owing to the large number of significant GO terms (biological process) in the green and pink modules, only the top 20 significant terms are displayed. Size and color of points represent -Log2 of FDR and number of genes associated with each term, respectively.

To investigate the putative functions associated with the modules, all the identified modules were subjected to functional enrichment analysis. Totally, 408 biological processes and 93 KEGG pathways were significantly enriched in 18 modules. Genes in the green module, as a highly preserved module, were significantly enriched in 49 and six biological processes and KEGG pathways, respectively. Only five KEGG pathways were enriched in the other highly preserved module, tan module. Also, 34, seven and seven biological processes and eight, five and no KEGG pathways were found in pink, lightyellow, and gray60 modules, respectively, as semi-preserved modules. The complete list of the functional enrichment analysis for highly and semi-preserved modules is available in Supplementary File S7. The top 20 significant biological process terms for highly and semi-preserved modules are presented in Figure 4.

**FIGURE 5:**
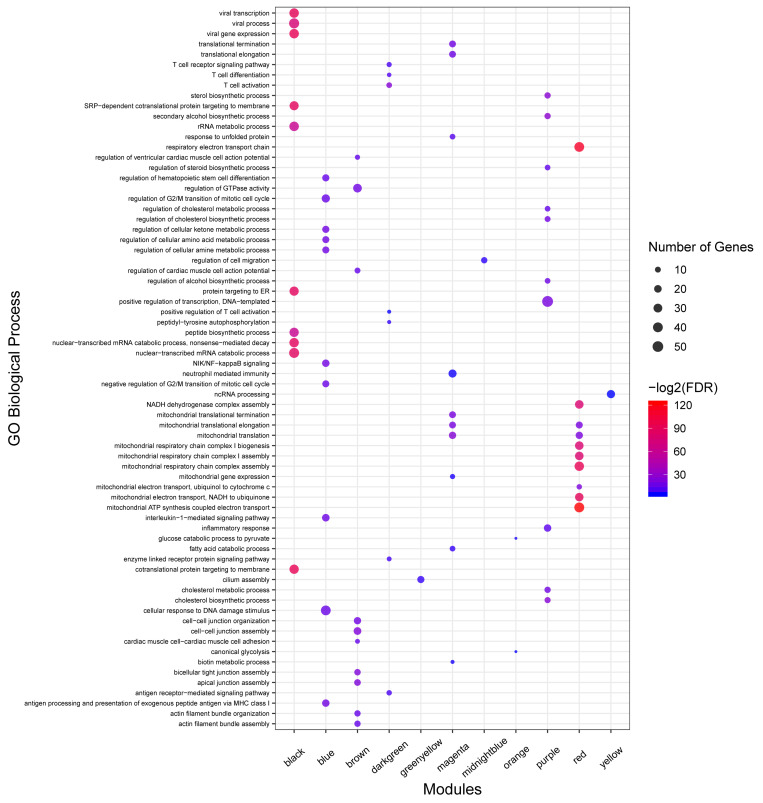
GO analysis results of non-preserved modules. Owing to the large number of significant GO terms (biological process) in blue, brown, red, magenta, black, and purple modules, only the top 10 significant terms are displayed. Size and color of points represent -Log2 of FDR and number of genes associated with each term, respectively. The fold enrichment measure is not shown for better visualization.

No enrichment of biological process and KEGG pathway were detected in seven non-preserved modules including lightcyan, cyan, darkgrey, darkred, darkturquoise, royalblue, and salmon modules. On the other hand, 312 biological processes and 67 KEGG pathways were significantly enriched in the other 13 non-preserved modules. Of these, eight non-preserved modules (including blue, brown, magenta, purple, darkgreen, red, midnightblue, and lightgreen) were associated with immune response functions based on the literature reports or term definition itself. The complete list of the functional enrichment analysis for non-preserved modules is available in Supplementary File S8. Moreover, the top 10 significant biological process terms for the eight non-preserved modules are presented in Figure 5.

### Hub Genes Identification in the Non-preserved Modules

**FIGURE 6:**
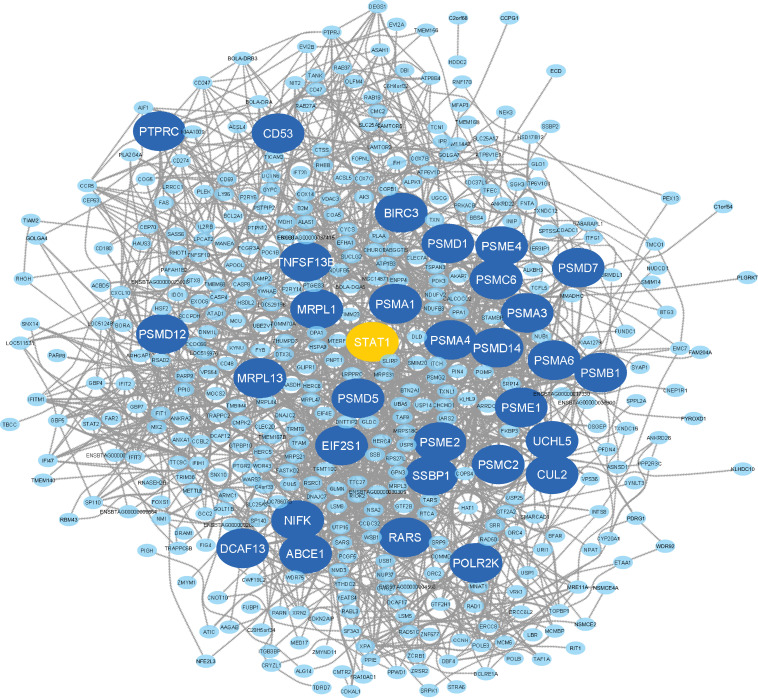
PPI network based on the hub genes of the blue module. The larger circles indicate highly connected genes and the orange circle represents the only TF among the highly connected genes.

Here, the eight non-preserved modules (blue, brown, magenta, purple, darkgreen, red, midnightblue, and lightgreen) were further examined to assess their topological behavior in terms of intra-modular connectivity to identify the hub genes. A total number of 533, 537, 234, 185, 47, 361, 69, and 88 hub genes were found in blue, brown, magenta, purple, darkgreen, red, midnightblue, and lightgreen non-preserved modules, respectively (Supplementary File S9). The identified hub genes in each module were analyzed for detecting significantly enriched biological processes and KEGG pathways. The results revealed 222 GO terms and 59 KEGG pathways similar to the functional enrichment analysis results of the modules where they have been assigned to (Supplementary File S10). In order to analyze the connections from the proteins encoded by the hub genes of each module, PPI networks were constructed for each hub gene sets in accordance with STRING database. The PPI networks of all eight hub gene sets showed significant connectivity. According to the important nodes detection approach described in the method section, 32, 32, 32, 36, 27, 32, 23, and 36 highly connected genes were detected in the constructed PPI networks, based on the hub genes in blue, brown, magenta, purple, darkgreen, red, midnightblue and lightgreen non-preserved modules, respectively (Table 1). These genes were hubs in their respective non-preserved modules and were also centrally located in their respective PPI networks, which make them promising candidates to illustrate the etiology behind bovine mastitis. The PPI network of the blue module is shown in Figure 6. Also, to have an overall view of the genes in the other non-preserved modules, their connections to each other (PPI networks) are provided in Supplementary File S11.

**TABLE 1:** List of the highly connected genes in the PPI networks that were constructed based on the hub genes of non-preserved modules.

Modules name							
Blue	Brown	Magenta	Purple	DarkGreen	Red	MidnightBlue	LightGreen
*PSMC6*	*MET*	*MRPL4*	*NFKBIZ*	*CD3E*	*NDUFAB1*	*PXN*	*WAS*
*PSMD14*	*ERBB3*	*ISCU*	*NR4A1*	*CD2*	*MDH2*	*VCL*	*LAT2*
*ITCH*	*CDH1*	*MRPL15*	*POLR2A*	*LCK*	*UQCRC1*	*PLCG2*	*MYO1F*
*UCHL5*	*JUP*	*MRPL19*	*CREBBP*	*ZAP70*	*COX6B1*	*TNS1*	*HCK*
*PSMD12*	*ERBB2*	*CAT*	*LDLR*	*ITK*	*UQCRC2*	*ACTN4*	*ARHGAP4*
*PSMA6*	*CLDN7*	*FECH*	*CXCL3*	*CD8A*	*NDUFA12*	*SPSB1*	*HMHA1*
*PSMA1*	*TJP1*	*EMG1*	*LPIN1*	*CD3G*	*NDUFC2*	*DGKD*	*VAV1*
*ABCE1*	*F11R*	*TXN2*	*GRO1*	*CD3D*	*UQCR10*	*GSN*	*TMC6*
*PSMA3*	*KRT5*	*PARK7*	*ABL1*	*CD52*	*CYC1*	*GRK6*	*KCNAB2*
*PSMA4*	*PROM1*	*GLRX5*	*RUNX1*	*TRAC*	*NDUFB6*	*CCR6*	*SYNGR2*
*PSME4*	*KRT8*	*GPX4*	*DAB2*	*LOC530077*	*UQCRFS1*	*PPAP2C*	*TMC8*
*PSME2*	*CCND1*	*COQ2*	*ACAT2*	*UBD*	*COX5A*	*IL1R1*	*MFNG*
*CUL2*	*ZEB1*	*MRPS27*	*NFKBIA*	*CD6*	*NDUFA9*	*ARSG*	*RASA4*
*PSMC2*	*FYN*	*PHB*	*ZC3H4*	*CXCR6*	*NDUFB10*	*XYLT1*	*SIRPA*
*PSMB1*	*EPCAM*	*SDHC*	*SREBF2*	*TCRB*	*NDUFA10*	*PRKCA*	*GLTSCR2*
*MRPL13*	*INADL*	*ACADM*	*DUSP2*	*PRF1*	*ACO2*	*CDH26*	*SH3PXD2B*
*PSMD1*	*CLDN4*	*GCLM*	*SRCAP*	*SKAP1*	*NDUFS6*	*SOCS2*	*TM9SF4*
*POLR2K*	*OCLN*	*ESD*	*TRRAP*	*GIMAP1*	*NDUFA6*	*THBS1*	*TMEM160*
*EIF2S1*	*CAV1*	*COX10*	*EPS15L1*	*LAMP3*	*UQCRB*	*BCL2L11*	*GSE1*
*PSME1*	*EZR*	*MRPL51*	*FOSB*	*CTSW*	*ISG15*	*BMF*	*SBNO2*
*PSMD5*	*LOC786683*	*CDK5*	*DUSP1*	*SH2D2A*	*NDUFS4*	*TMEM201*	*BCL3*
*NIFK*	*YAP1*	*SORD*	*RARA*	*GIMAP4*	*MRPL21*	*TIAM1*	*NEURL1*
*PSMD7*	*RHOC*	*MRPS30*	*FOXF1*	*IL16*	*NDUFA2*	*IL1RAP*	*KIF21B*
*BIRC3*	*CGN*	*AHSA2*	*PLB1*	*CST7*	*IDH3B*		*PSD4*
*SSBP1*	*FHL2*	*SIL1*	*PRDM2*	*GFI1*	*NDUFS7*		*DTX2*
*MRPL1*	*PARD3*	*ETFDH*	*CXCL2*	*LY6E*	*COX7A2*		*KCNT1*
*DCAF13*	*DSG2*	*CDK5RAP1*	*FOXO1*		*NDUFA13*		*ADRBK1*
*STAT1*	*KRT18*	*HSPA4L*	*SIK1*		*NEDD8*		*SIPA1*
*PTPRC*	*DSP*	*HCCS*	*ACSS2*		*COX6A1*		*ARRB2*
*TNFSF13B*	*KRT7*	*AMACR*	*CREB3L2*		*DDOST*		*SCARF1*
*RARS*	*DOCK5*	*ALS2*	*SMURF1*		*ACSF2*		*CARD9*
*CD53*	*FOXA1*	*CD68*	*SSTR2*		*COX7A2L*		*PWP2*
			*FLNA*				*PREX1*
			*LSS*				*NOL4L*
			*MAMLD1*				*CYB5R1*
			*PHF12*				*POR*

## Discussion

Mastitis remains among the most challenging disorders in the cattle industry to treat. In this study, WGCNA approach was utilized to improve the efficiency of important genes identification through clustering the genes into modules that are likely enriched for particular biological pathways associated with bovine mastitis. The genes in 72% of the identified modules (18 out of 25) were significantly enriched in at least one GO term or KEGG pathway, which indicated that signed WGCNA effectively clustered the co-regulated and biologically related genes into separate modules. Functional enrichment analysis of the preserved and semi-preserved modules showed enriched terms representing basic biological processes such as “translation,” “rRNA modification,” and “DNA replication.” Therefore, based on the preservation structure and functional enrichment results, these modules cannot potentially distinguish infected from healthy samples. On the other hand, gray60 and lightyellow modules (as semi-preserved) showed some terms that are potentially related to mastitis such as “neutrophil mediated immunity” and “inflammatory response.” However, we focused on the eight non-preserved modules (blue, brown, magenta, purple, darkgreen, red, midnightblue, and lightgreen), which exhibited significantly altered intramodular connectivity in the infected samples as well as showed functional terms related to mastitis or immune responses. It is worth to note that various pathogens, which can cause mastitis, induce different immune responses in the bovine mammary epithelial cells (Wellnitz and Bruckmaier, 2012). Subsequently, we narrowed down the list of genes within the non-preserved modules by focusing on the hub genes. The constructed PPI networks based on the hub genes of all the eight non-preserved modules showed a significant and ideal connectivity, which emphasized effectiveness of our method to organize functional modules that comprises of a set of proteins having similar functions. Hence, these modules might influence the pathogenesis process of mastitis and, therefore, warrant further validation.

Based on the enriched functional terms in the blue module, which were potentially related to mastitis development, this module was determined as one of the most important modules in the present study. Some of the enriched terms included “innate immune response,” “*NIK/NF-kappaB* signaling,” “interleukin-1-mediated signaling pathway,” “NOD-like receptor signaling pathway,” “toll-like receptor 4 signaling pathway,” “neutrophil mediated immunity,” “T cell receptor signaling pathway,” “cytokine-mediated signaling pathway,” “regulation of defense response,” and “response to cytokine.” Pathogens invades mammary epithelial cells through pattern recognition receptors, which induce different signaling pathways and lead to the establishment of an inflammatory response. Toll-like receptors (*TLRs*) are one of the most important pattern recognition receptors in various cell types (Dinarello, 2018). *TLRs* are key participants in the induction of innate immune responses in the mammary gland cells through recognizing various bacterial cell wall components such as lipopolysaccharide (*LPS*, typical of gram-negative bacteria, e.g., *Streptococcus uberis*) and lipoteichoic acid (*LTA*, typical of gram-positive bacteria, e.g., *Staphylococcus aureus*) (Aderem and Ulevitch, 2000; Taraktsoglou et al., 2011). In this regard, activation of *TLR4* is linked to the expression of pro-inflammatory cytokines and the activation of *NF*-*kappaB* signaling pathway in mastitis (Wu et al., 2015). Upregulation of *TLR4* in bovine macrophages after stimulation with either *LTA* or *LPS* has been demonstrated (Franchini et al., 2006). *NF-kappaB* signaling pathway is a key pathway responsible for the expression of pro-inflammatory cytokines (Wu et al., 2016). This pathway modulates the expression of many inflammation-related genes (such as inflammatory cytokines *TNF-*α, *IL-1*β, and *IL-6*), especially in mastitis (Heyninck et al., 2014). Interestingly, in our study, *TLR4* was detected as the hub gene in the blue module. One of the other important pattern recognition receptors are interleukin-1 receptors and their important functions in acute bacterial mastitis have been documented (Filipe et al., 2019). Also, the higher expression of interleukin family members is reported during mastitis infection (Lutzow et al., 2008; Compton et al., 2009). NOD-like receptor signaling pathway is responsible for detecting various pathogens and mediate numerous aspects of innate immunity (dan Xu et al., 2017). This pathway acts in parallel with the Toll-like receptor signaling pathway (Aderem and Ulevitch, 2000). Additionally, NOD-like receptor signaling pathway was observed among the 30 most impacted pathways in the three gene expression-based studies on bovine mastitis (Loor et al., 2011). Neutrophils as a source of small antibacterial peptides, are considered to be a primary defense mechanism to kill a variety of mastitis-causing pathogens. They are the predominant cell types found in the mammary glands during early stage of mastitis and recognize, adhere, and phagocyte invading pathogens (Medina, 2009). T cell receptor signaling pathway is related to adaptive immunity and has been reported as a candidate pathway associated with occurrence and development of mastitis in dairy cow (Luoreng et al., 2018).

Additionally, in terms of individual highly connected genes identified in the blue module, several genes such as *BIRC3, PSMA6, PSMB1, PSMD12, PSMD14*, *PSMD7* (Brand et al., 2011; Loor et al., 2011), *EIF2S1* (Appuhamy et al., 2011), *PTPRC* (Nicholas et al., 2003), and *CD53* (Rinaldi et al., 2010) have been reported as important genes in mastitis development. Among the highly connected genes of the blue module, *STAT1* was the only TF (Figure 6 and Supplementary File S12). The gene members of the signal transducers and activators of transcription (*STAT*) family (including *STAT1*, -2, -3, -4, -5a, -5b, and -6) have been reported as important factors involved in every stage of mammary gland development (Philp et al., 1996; Cobanoglu et al., 2006). Of these, *STAT3* and *STAT5* have been well-known as important regulators during mammary gland development and tumorigenesis (Philp et al., 1996). The functions of *STAT1*, *STAT3*, *STAT5*, and *STAT6* in breast cancer formation, progression, prognosis and prediction have been documented (Haricharan and Li, 2014). Moreover, *STAT3* has recently been reported as a potential therapeutic target in mastitis. In this regard, *STAT1* has been shown to be important in immune cells in mastitis (Hughes and Wood, 2017). On the other hand, the role of *STAT1* as a tumor suppressor has been demonstrated in human breast cancer (Haricharan and Li, 2014). In a good agreement with the recent studies that consider *STAT* family members as more important candidates in mammary gland development, in the present study, *STAT1* was found as the only highly connected TF in the blue module, which reinforce the potential function of this regulator in defense against infection-causing bacteria. Since, co-expressed genes are likely to be functionally related and regulated by the same TF (Behdani and Bakhtiarizadeh, 2017; Bakhtiarizadeh et al., 2018), the other highly connected genes in the blue module might be ideal candidates to better understand molecular mechanisms behind mastitis.

Genes in the brown module showed KEGG pathway enrichment in “Leukocyte transendothelial migration,” “Cell adhesion molecules,” and “Focal adhesion.” Focal adhesion is necessary for cell migration and some important biological processes such as leukocyte transendothelial migration (Jin et al., 2016; Chang et al., 2017). Leukocyte transendothelial migration is an important process in inflammation and the innate immune system that cause the first cellular responders to migrate into infected tissue (Getter et al., 2019). A huge influx of polymorphonuclear leukocytes (the major leukocyte cell type) into the infected mammary glands is an initial inflammatory response to bacterial infection (such as mastitis) (Shah et al., 2018), where they may combat invading pathogens. Cell adhesion molecule is the other important part of the host immune system. Interaction between leukocytes and specific endothelial cell adhesion molecules has been demonstrated, which helps to regulate the migration of leukocytes to the site of inflammation (Kleczkowski et al., 2017). Additionally, some of the highly connected genes belonging to this module have been found by others to be related to mastitis development, immune response or mammary gland development including *FYN, CDH11, CAV1, F11R, ZEB1, ERBB2*, and *ERBB3* (Cohen et al., 2015; Yang et al., 2018). For example, *F11R* was reported as a candidate gene of mammary gland immune response (Kosciuczuk et al., 2017). These findings supporting the potential functions of the brown module during mastitis infection.

Enrichment of “*TNF* signaling pathway,” “*NF-kappa B* signaling pathway,” “Toll-like receptor signaling pathway,” and “*MAPK* signaling pathway” in the purple module revealed the potential functions of its members in mastitis pathogenesis. Tumor necrosis factor (*TNF*) is of the cytokine family that coordinates the mammalian immune response and secrete by macrophages in response to endotoxins. *TNF* signaling pathway is a classical immune system pathway, which has a central role in the control of inflammation, immunity and cell survival (Galvão et al., 2012; Rana et al., 2019). Hence, it is suggested that a potential mechanism to block the development of inflammation, by the effective medicines that used for treating mastitis, is inhibiting *TNF* signaling pathway through reducing the secretion of *TNF* (Wu et al., 2019). *MAPK* signaling pathway plays a key role during inflammatory responses (Kim et al., 2014). It is well-accepted that both of *MAPK* and *NF-kappaB* signaling pathways can induce the expressions of various inflammatory mediators and pro-inflammatory cytokines. In a previous study, both of these pathways were activated in *LPS*-induced mastitis (Liang et al., 2014). Some of the highly connected genes in the purple module have previously been related to mastitis development including *NFKBIZ* (Compton et al., 2009), *NFKBIA, CXCL2, GRO1* (Li et al., 2019), *CXCL3* (Rainard et al., 2008), *LPIN1* (Moyes et al., 2009), and *DAB2* (Banos et al., 2017). For example, *NFKBIZ* is a regulator of *NF-kappaB* and gene variants of this gene is introduced as potential markers of mastitis resistance in dairy heifers (Compton et al., 2009). These results suggested that the members of this module may contribute to the pathogenesis of mastitis.

In the magenta module, genes with annotated functions in “neutrophil activation involved in immune response” and “neutrophil mediated immunity” were enriched and are likely to be related to mastitis. Among the highly connected genes in the magenta module, *GCLM* (Wang K. et al., 2016), *PARK7* (Donaldson et al., 2005), *SIL1* (Li et al., 2019), *PHB* (Genini et al., 2011), and *CD68* (Bilir et al., 2012) were potentially associated with mastitis or similar biological processes, based on the previous studies. For instance, *PARK7* has been shown to play an important role in the bovine immune response (Donaldson et al., 2005).

In agreement with the previous studies (Genini et al., 2011; Luoreng et al., 2018), the functional enrichment results of the darkgreen module reinforced that the “T cell receptor signaling pathway” might be involved in mastitis development. Accordingly, *CD3E* (Paquette et al., 2015), *CD2* (Rivas et al., 2002), *LCK* (Nie et al., 2012), *ZAP70* (Schulman et al., 2009), *CD3D* (Bonnefont et al., 2011), *PRF1* (Twigger et al., 2018), *CD8A* (Kosciuczuk et al., 2017), and *IL16* (Sharmila et al., 2002) were identified as highly connected genes of this module and also have been reported as important genes in immune response or mastitis development.

Genes with biological processes enrichment in “cytokine-mediated signaling pathway” was observed in the red module. It is well-known that the transcription and secretion of proinflammatory/regulatory cytokines occur during the stimulation of bovine macrophages with *LPS* (in gram-negative bacteria, e.g., *Streptococcus uberis)* or *LTA* (in gram-positive bacteria, e.g., *Staphylococcus aureus*). The inflammatory cytokines can activate the host defense mechanisms during mastitis (Aderem and Ulevitch, 2000; Taraktsoglou et al., 2011). *TLR6*, as a hub gene in the red module, is an essential component of the recognition complex for *LTA* (Henneke et al., 2005).

The important enriched pathways in midnightblue module were “Leukocyte transendothelial migration” and “Inflammatory mediator regulation of *TRP* channels.” Transient receptor potential (*TRP*) channels are important mediators of sensory signals that have contribution in the detection of physical stimuli including inflammatory stimulations (Levine and Alessandri-Haber, 2007; Zielińska et al., 2015). In the case of the midnightblue module, some of the highly connected genes including *GSN* (Polgar et al., 2012), *CCR6* (Polgar et al., 2012), *SOCS2* (Rupp et al., 2015), *THBS1* (Wang X.G. et al., 2016), *IL1R1*, and *IL1RAP* (Bonnefont et al., 2011) were involved in mastitis defense or immune response. For example, *GSN* (Marchitelli et al., 2017) has been suggested as indicator of mastitis and many studies have documented important function of *SOCS2* in the regulation of cytokines and mastitis (Bonnefont et al., 2011).

In the lightgreen module, KEGG pathway analysis of the genes indicated that the “Chemokine signaling pathway” and “Fc gamma R-mediated phagocytosis” were significantly enriched, which are part of the immune system. These pathways were also found to be significantly enriched in the up-regulated genes in the mammary gland of dairy cattle with *E. coli*-induced mastitis (Buitenhuis et al., 2011). In this module, the most important highly connected genes were *HCK*, which encodes two isoforms of a protein-tyrosine kinase involved in mammary gland immune response (Kosciuczuk et al., 2017) and *VAV1*, which encodes a unique protein involved in tyrosine-mediated signal transduction and immune response (Turner and Billadeau, 2002).

## Conclusion

Our results led to identify the eight modules of genes, which were non-preserved between the healthy and infected samples and may play important roles in the pathogenesis of mastitis. Integration of the co-expression network with PPI data enabled us to identify highly connected genes that were hubs in both co-expression and PPI networks. Totally, based on our topological and functional analysis of the eight non-preserved modules, 250 highly connected genes were found, as most of them were directly or indirectly associated with mastitis. These genes can be considered as potential targets for future research aimed at understanding the function of important genes in mastitis pathogenesis. Moreover, the other members of the eight non-preserved modules may potentially play important roles in mastitis development. Therefore, a further analysis and validation of the candidate genes reported here deserve further study, including those that have not yet been associated with mastitis or immune response.

## Data Availability Statement

RNA-Seq data of healthy and infected bovine samples were obtained from the Gene Expression Omnibus (GEO) database at the National Center for Biotechnology Information (NCBI) under accession number of GSE51858.

## Author Contributions

MB conceived the ideas. MB and SM designed the study and analyzed the data. MB, MN, NS, and MV interpreted the data and wrote the main manuscript text. All authors contributed to the article and approved the submitted version.

## Conflict of Interest

The authors declare that the research was conducted in the absence of any commercial or financial relationships that could be construed as a potential conflict of interest.

## Supplementary Material

FILE S1 Sample clustering to detect outliers. All samples (except tc13) were well-clustered.

FILE S2 Analysis of network topology for a set of soft thresholding powers. The left plot displays the scale of free fit index (*y*-axis) as a function of the soft thresholding power (*x*-axis). The right plot shows the mean connectivity (degree, *y*-axis) as a function of the soft thresholding power (*x*-axis).

FILE S3 Descriptive statistics of sequence quality and mapping rate of the RNA-Seq data.

FILE S4 The gene expression matrix of all samples in normal and mastitis groups.

FILE S5 The list of genes in each module.

FILE S6 Summary of module preservation analysis.

FILE S7 The complete list of the functional enrichment analysis for highly and semi-preserved modules.

FILE S8 The complete list of the functional enrichment analysis of the identified non-preserved modules.

FILE S9 The complete list of the hub genes in each module.

FILE S10 The complete list of the functional enrichment analysis of the hub genes in the eight non-preserved modules.

FILE S11 Protein-protein interaction networks based on the hub genes of the non-preserved modules of interest.

FILE S12 The complete list of transcription factors per module. The genes of the modules were aligned with bovine transcription factors extracted from AnimalTFDB database (http://bioinfo.life.hust.edu.cn/AnimalTFDB/).
