# Supplementary material for: Identification of Gene Modules and Hub Genes Involved in Mastitis Development Using a Systems Biology Approach
Source: Front Genet. 2020 Jul 13;11:722. doi: 10.3389/fgene.2020.00722 (PMC7371005; doi:10.3389/fgene.2020.00722)
Supplement: FILE S11 — Protein-protein interaction networks based on the hub genes of the non-preserved modules of interest. [file Table_11.DOCX]

**Integrative network analysis identifies functional modules and highly connected genes in mastitis development**

Mohammad Reza Bakhtiarizadeh*, Shabnam Mirzaei, Milad Norouzi^1^, Negin Sheybani^1^, Mohammad Sadegh Vafaei sadi^1^

Department of Animal and Poultry Science, College of Aburaihan, University of Tehran, Tehran, Iran.

^1^ These authors contributed equally in this study

* Corresponding author. E-mail address: [mrbakhtiari@ut.ac.ir](mailto:mrbakhtiari@ut.ac.ir), ORCID ID: ​0000-0001-5336-6987

**Supplementary File S11:** Protein-protein interaction networks based on the hub genes of the non-preserved modules of interest.


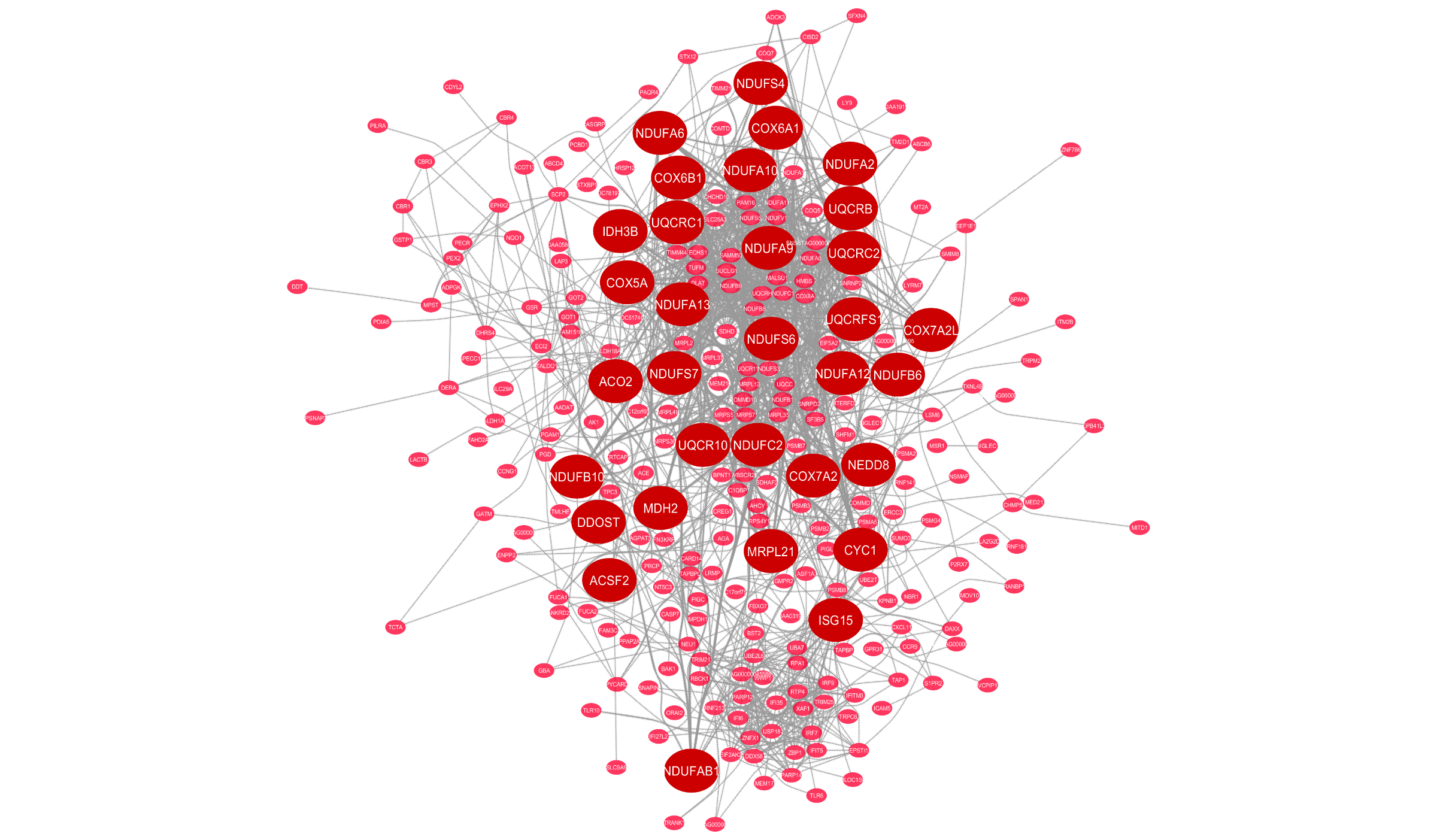


**Red module**


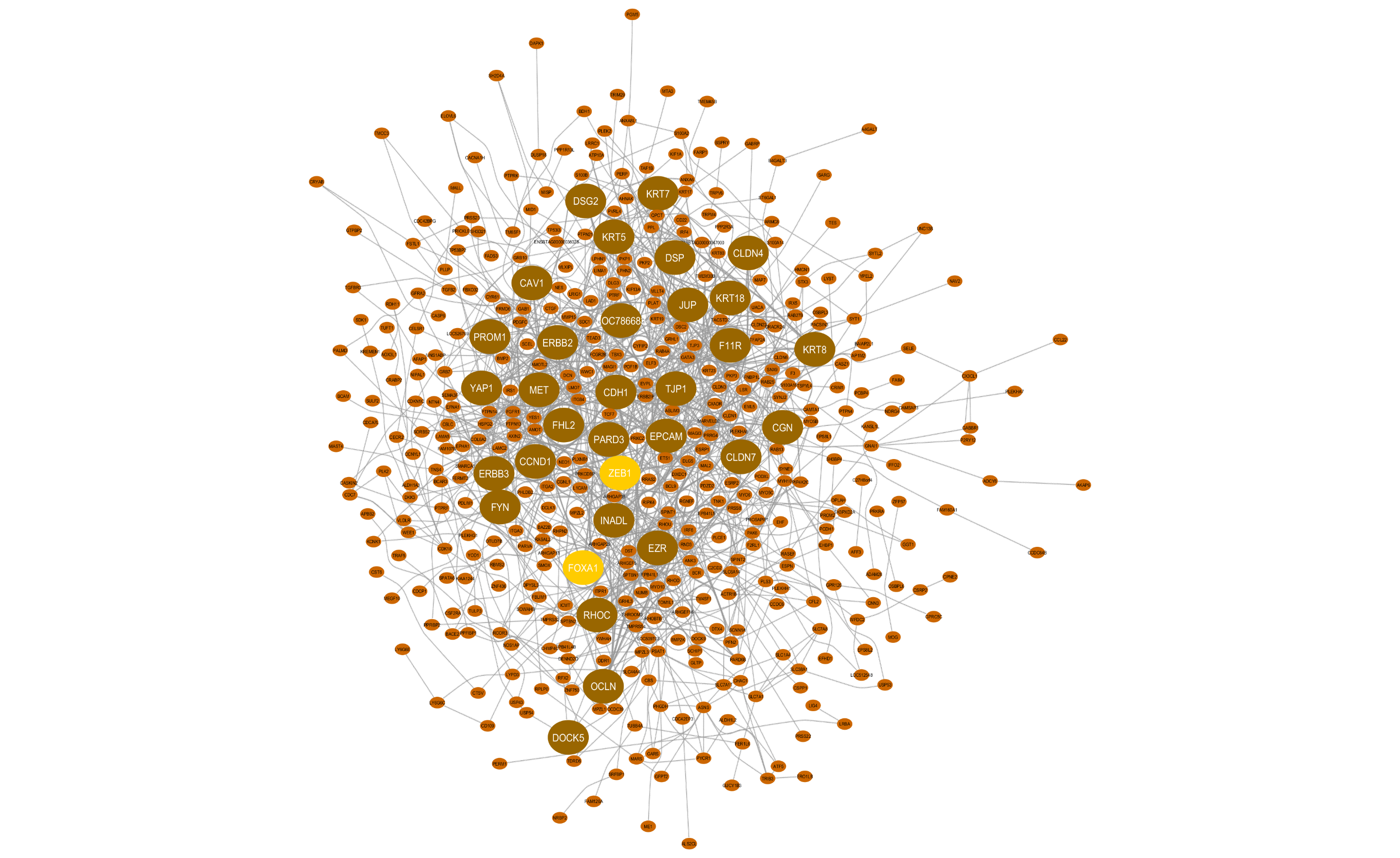


**Brown module**


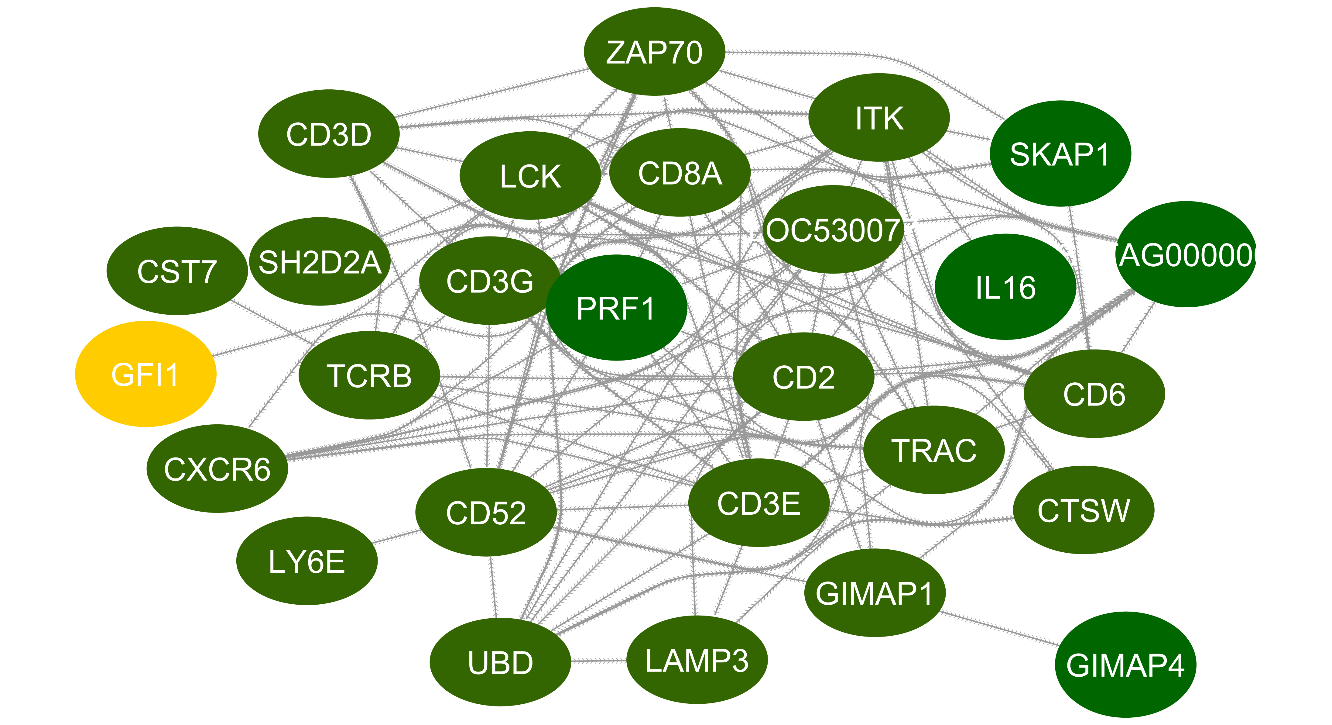


**Darkgreen module**


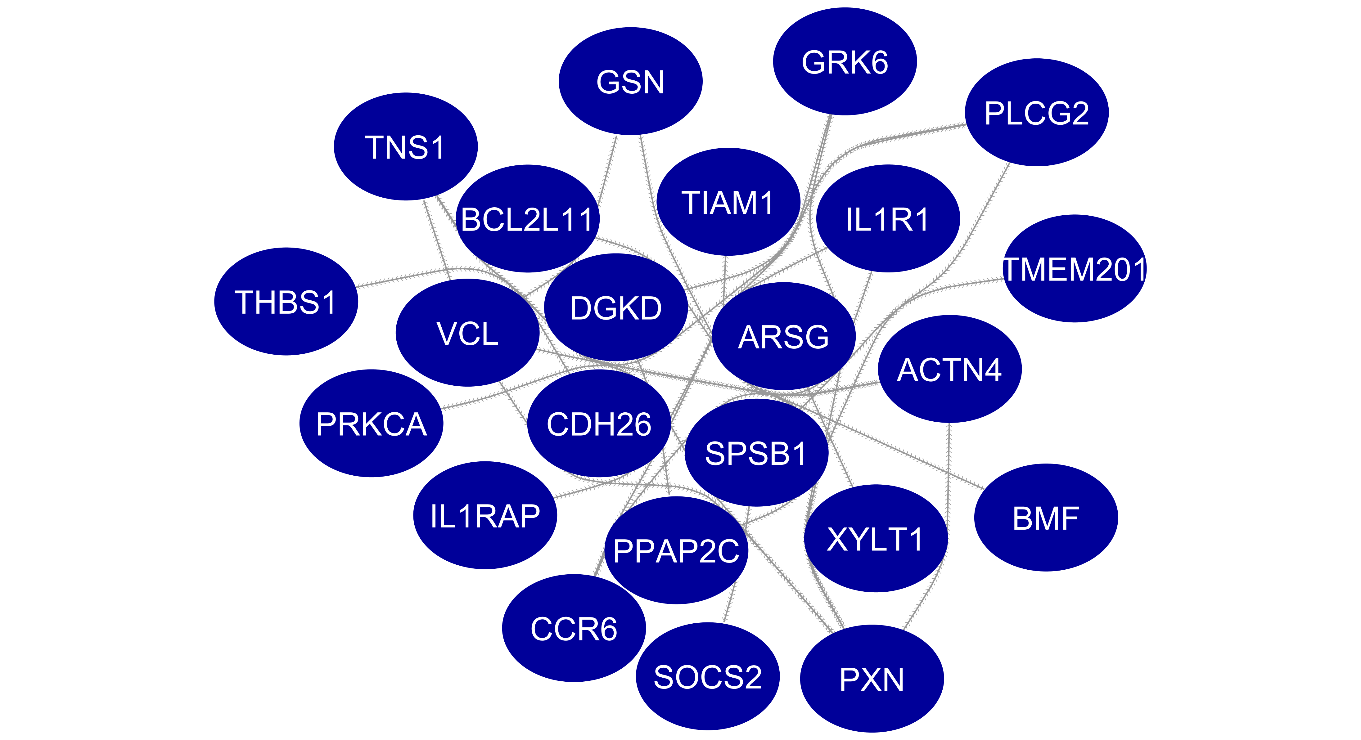


**Midnight blue**


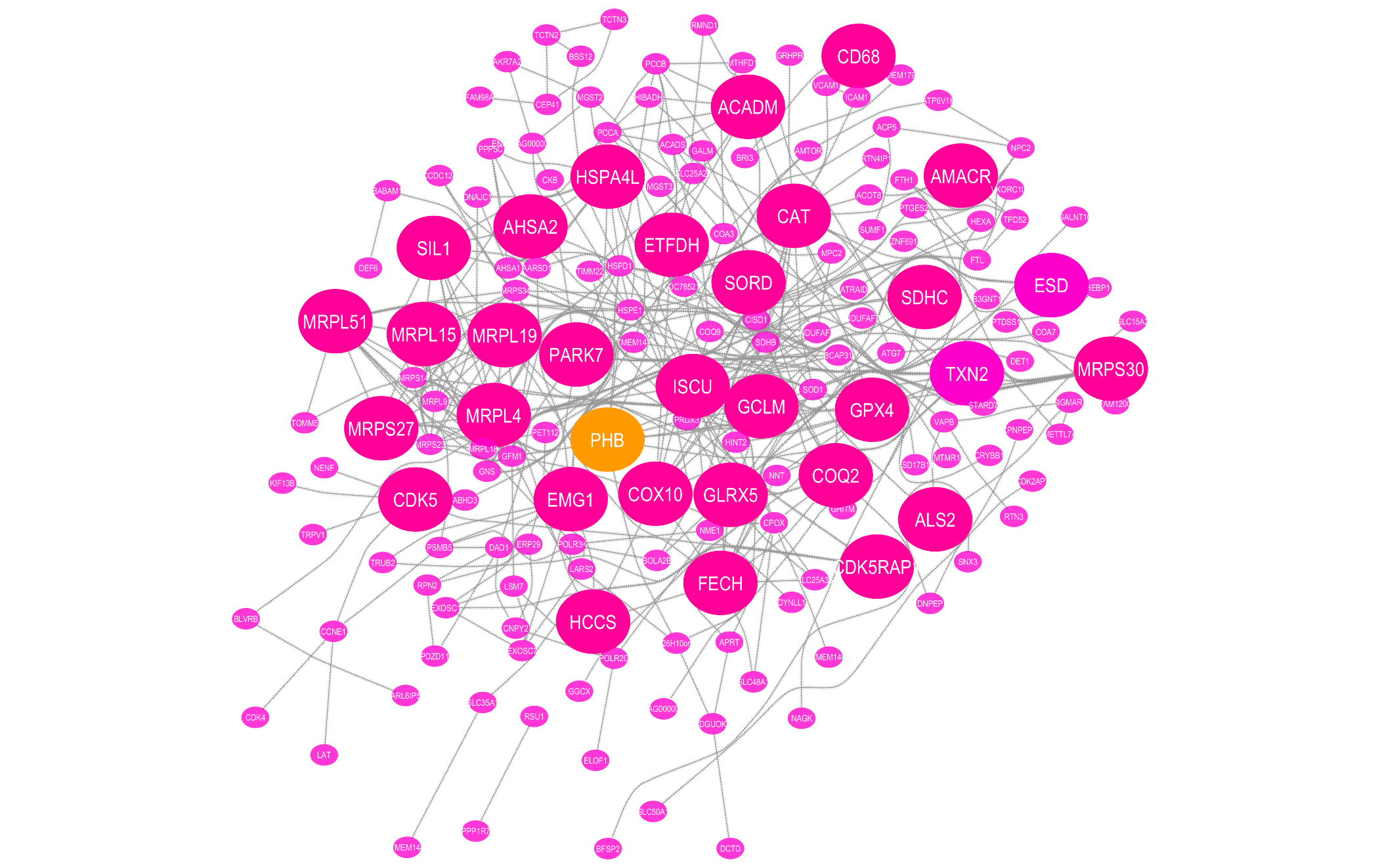


**Magenta module**


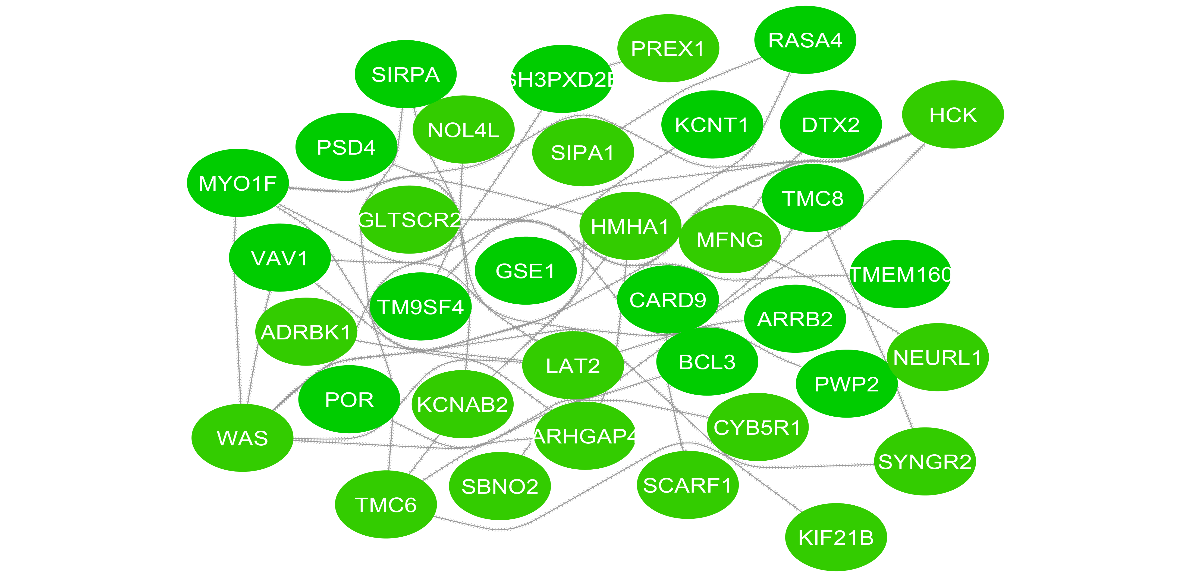


**Lightgreen module**


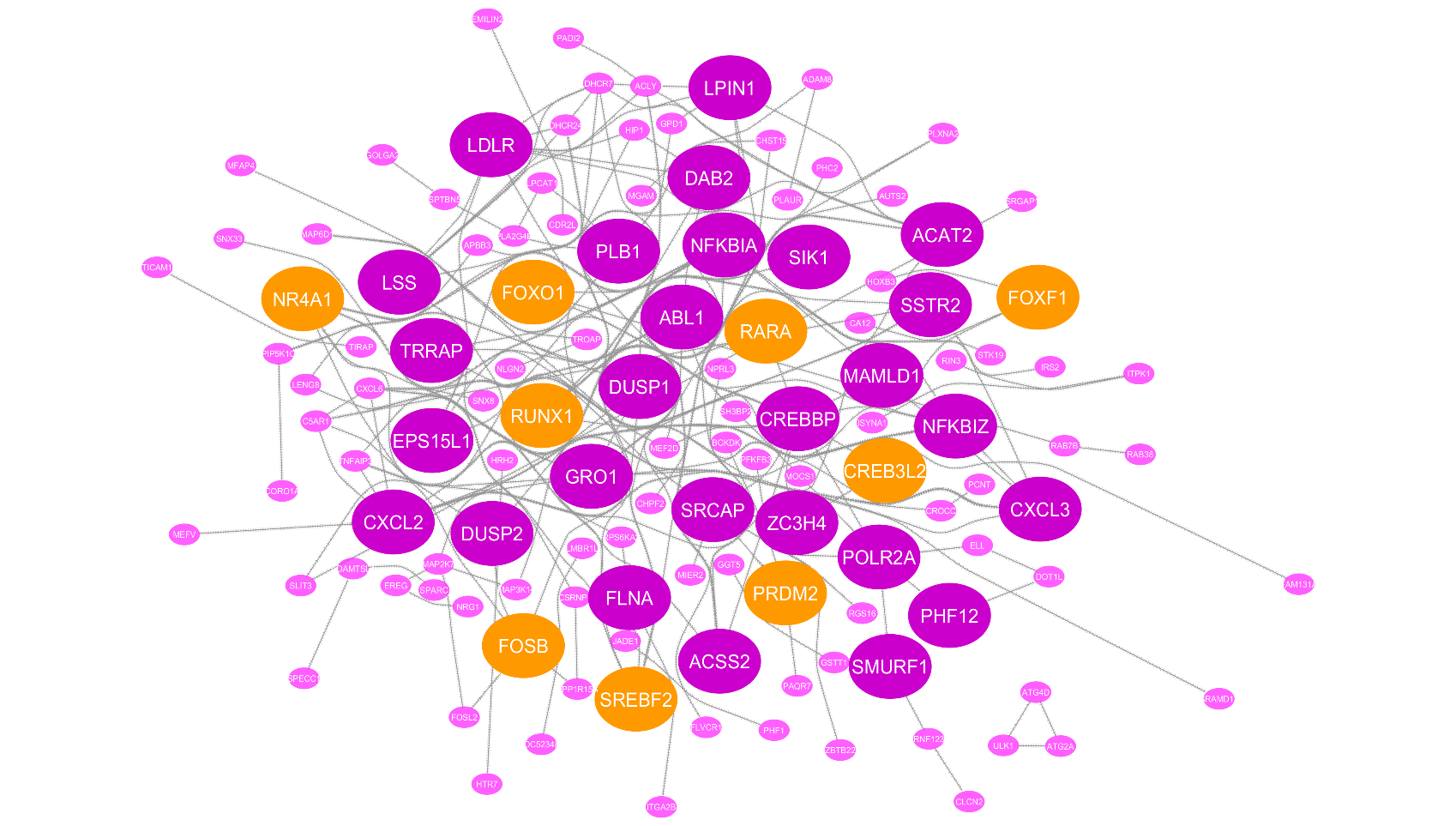


**Purple** **module**
